# Exploring Marchiafava-Bignami Disease: A Rare Presentation in a Young Pregnant Female

**DOI:** 10.7759/cureus.61701

**Published:** 2024-06-04

**Authors:** Abhinav Kadam, Saket S Toshniwal, Jiwan Kinkar, Sunil Kumar, Sourya Acharya

**Affiliations:** 1 Department of Medicine, Jawaharlal Nehru Medical College, Datta Meghe Institute of Higher Education and Research, Wardha, IND; 2 Department of Neurology, Jawaharlal Nehru Medical College, Datta Meghe Institute of Higher Education and Research, Wardha, IND

**Keywords:** parenteral thiamine, marchiafava-bignami disease, corpus callosum, hyperemesis gravidarum, demyelinating neurological disorder

## Abstract

Marchiafava-Bignami disease (MBD) is uncommon and typically linked with persistent alcohol consumption; nevertheless, instances have been reported in non-alcoholic individuals with nutritional deficiencies. Depending on the severity, this condition may manifest as acute, subacute, or chronic neurological signs and symptoms, ranging from moderate dysarthria or mild disorientation to coma and death. We report a case of a 30-year-old, 14-week pregnant female who presented with complaints of persistent vomiting and loss of appetite. She was found to have achalasia cardia on upper GI endoscopy. Later, she developed confusion, irrelevant talks and her speech was incomprehensible. An MRI of the brain was done which showed features likely that of MBD. She was started high dose intravenous thiamine to which she responded brilliantly.

## Introduction

Marchiafava-Bignami disease (MBD) is an uncommon disorder linked with persistent drinking, characterized by demyelination or necrosis of the corpus callosum (CC) [1]. In certain cases, the damage even spreads to the white matter of the hemisphere [2]. Male patients between the ages of 40 and 60 who have a history of malnourishment and persistent drinking are the majority affected by MBD. Clinical diagnosis is challenging and depends on imaging, especially magnetic resonance imaging (MRI) to show disease characteristics [3]. The signature of the condition is brain MRI demonstrating the involvement of the CC. However, there are many different presenting patterns in the clinical presentation and no particular or pathognomonic clinical symptoms are present. We present a case of a 30-year-old pregnant female who developed MBD as a consequence of chronic malnutrition precipitated by Hyperemesis gravidarum.

## Case presentation

A 30-year-old female, G2L1 with a gestation age of 14 weeks, presented to this hospital with a history of decreased oral acceptance and persistent vomiting for one month. She stated regular visits to an obstetrician with the ultrasonographic assessment of fetal growth two times during antenatal care (ANC) duration. She had a history of sudden pain in the abdomen 15 days ago for which she was taken to a general surgeon to search for the cause of abdominal pain. Ultrasonography of the abdomen and fetal screening were done at that time which showed intrauterine death of the fetus. An obstetrician was consulted and the fetus was expelled out of the uterus by means of induction of labor with Tab. Misoprostol. She was discharged within two days with antibiotics and analgesic medication on discharge. The patient did not have any comorbidity and did not take any medication daily. On examination, the patient's pulse was 94 beats per minute (bpm), blood pressure was 130/80 mmHg, and oxygen saturation was 99% on room air. There were no signs of pallor, icterus, clubbing, cyanosis, lymphadenopathy, or pedal edema. On systemic examination, the cardiovascular and respiratory system examination was within normal limits. The abdomen examination did not reveal anything significant, apart from appropriate findings due to a gravid uterus. Central nervous system examination revealed no significant abnormality. She was admitted to the female medicine ward for intravenous fluid administration and control of vomiting episodes.

All biochemical and pathological investigations were sent, which came back as described in Table 1.

**Table 1:** Laboratory investigations of the patient on admission g/dl: gram per decilitre; micron: micrometer; pg: picogram; cumm: cubic millimeter; fL: femtolitre; mg/dl: milligrams/decilitre; mEq/l: milliequivalents/liter; IU/L: international units/liter; IU/ml: international units/milliliter; U/L: units/liter; mm/Hr: millimeter/hour; mg/L: milligrams/liter; MCHC: mean corpuscular hemoglobin concentration; MCV: mean corpuscular volume; MCH: mean corpuscular hemoglobin; RBC: red blood corpuscles; WBC: white blood cell; RDW: red cell distribution width; APTT: activated partial thromboplastin time; INR: international normalized ratio; SGOT: serum glutamic oxaloacetic transaminase; SGPT: serum glutamic pyruvic transaminase; HIV: human immunodeficiency virus

Laboratory parameter	Results	Normal values
Hemoglobin	10.1 g/dl	11-14 g/dl
MCHC	32.2 g/dl	32-36 g/dl
MCV	96 micron	79-92 micron
MCH	31.5 pg	27-31 pg
Total RBC count	3.51 x 10^6 ^cells/cumm	2.50-5.50 x 10^6 ^cells/cumm
Total WBC count	9300 cells/cumm	4000-11000 cells/cumm
Total platelet count	2.72 x 10^6 ^cells/cumm	1.50-4.50 x 10^6 ^cells/cumm
Hematocrit	39.6%	40-54%
Monocyte	3%	2-8%
Granulocyte	56%	40-60%
RDW	14.8 fL	12.2-16.1 fL
Eosinophils	1%	1-4%
Basophil	0%	<1%
APTT	30.2 seconds	29.5 seconds
Prothrombin time	11.7 seconds	11.3 seconds
INR	1.13	1.00
Urea	11 mg/dl	6-24 mg/dl
Creatinine	0.6 mg/dl	0.59-1.04 mg/dl
Sodium	137 mEq/l	135-145 mEq/l
Potassium	3.0 mEq/l	3.5-5.1 mEq/l
Alkaline phosphate	78 IU/L	75-124 IU/L
SGOT	48 IU/L	8-45 IU/L
SGPT	43 IU/L	7-56 IU/L
Total protein	7.9 g/dl	6.0-8.3 g/dl
Albumin	4.1 g/dl	3.4-5.4 g/dl
Total bilirubin	0.9 mg/dl	0.1-1.0 mg/dl
Conjugated bilirubin	0.2 mg/dl	0.1-0.4 mg/dl
unconjugated bilirubin	0.7 mg/dl	0.2-0.6 mg/dl
HIV card test	Negative	-

In view of persistent vomiting, an upper gastrointestinal endoscopy was done to look for the cause. It showed a fluid and food-filled lower esophagus suggestive of achalasia cardia. She was planned for further intervention to manage achalasia. On the fourth day since admission, she started becoming confused, talking irrelevant and her speech was incomprehensible. She was admitted under neurology in view of altered sensorium. A couple of advanced and invasive investigations were done to rule out endocrinological, autoimmune, or infective etiology, as shown in Table 2.

**Table 2:** Laboratory investigations of the patient on the fourth day since admission uIU/ml: micro-international units/milliliter; ng/dl: nanograms/deciliter; mg/dl: milligrams/deciliter; mmol/l: millimole/liter; cells/microL: cells/microliter; TSH: thyroid-stimulating hormone; T3: triiodothyronine; T4: thyroxine; ANA: antinuclear antibody

Laboratory parameter	Results	Normal values
TSH	3.31 uIU/ML	0.465-4.68 uIU/ML
T3	1.24 ng/ml	0.970-1.69 ng/ml
T4	6.35 ug/dl	5.53-11.0 ug/dl
ANA	0.7	<0.9
CSF STUDY	Suggestive of 2 ml of clear fluid, containing glucose - 78 mg/dl, protein - 24 mg/dl, lactate - 1.7 mmol/l, TLC - 4 cells/microL, lymphocytes - 88%, monocytes - 6%, neutrophils - 6%, showed no growth after 72 hours of incubation.	

**Figure 1:**
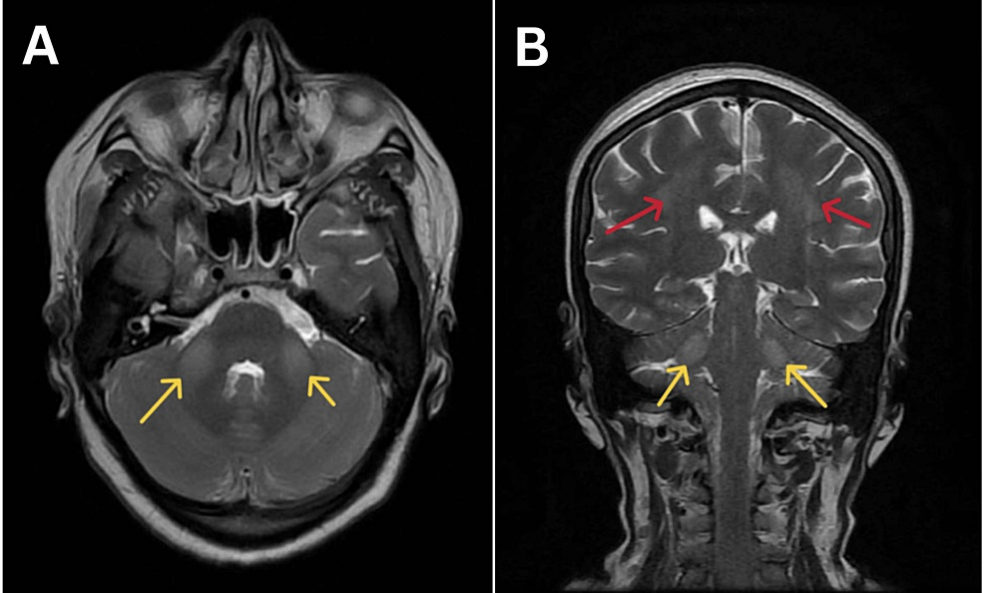
Magnetic resonance imaging of the brain of the patient suggestive of flair sequences showing hyperintensities in the middle cerebral peduncle (yellow arrows) and hyperintensities in parietal lobes (red arrows); A) axial section B) coronal section

**Figure 2:**
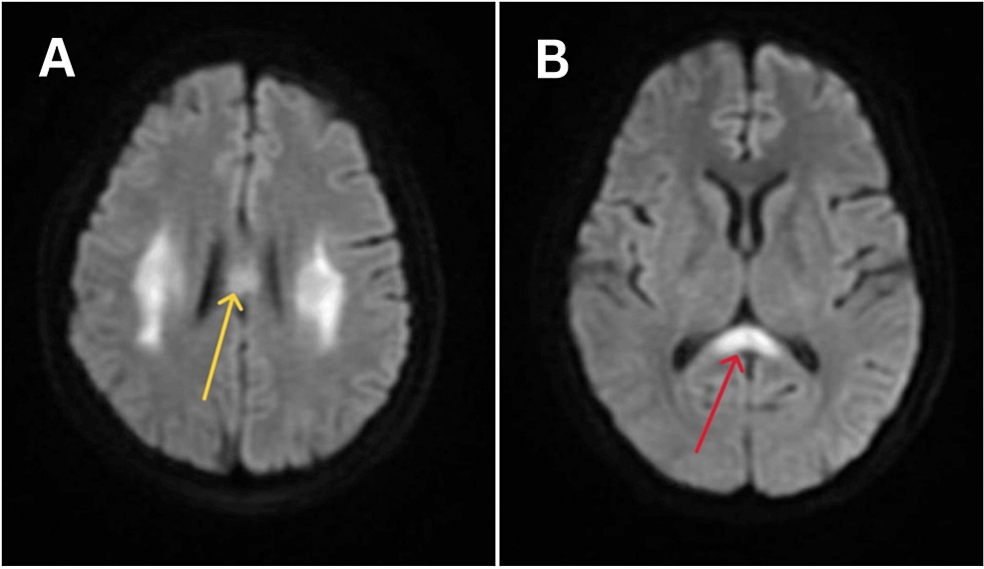
Magnetic resonance imaging of the brain of the patient suggestive of diffusion weighted-imaging sequences of the axial section showing A) hyperintensities in the body of the corpus callosum (yellow arrows) B) hyperintensities in parietal lobes (red arrows)

MRI brain with contrast showed bilateral middle cerebral peduncle (MCP) hyperintensities, splenium hyperintensities, and high parietal hyperintensities (Figures 1, 2).

In view of her chronic nutritional deprived state, persistent vomiting and altered sensorium paired with MRI findings, a possibility of Marchiafava Bignami disease was suspected. She was started on a high dose of injection of Thiamine 500 mg IV every 24 hours for three days, followed by 100 mg 8 hourly for the next seven days and other supportive management. She started to respond to this treatment immediately. Her sensorium improved, and she became oriented to time, place, and person within 24 hours after Thiamine administration. She was kept in the hospital for observation for the next seven days and was discharged within a total of 10 days of hospital stay.

## Discussion

Two Italian pathologists first defined MBD after finding deterioration of the CC in three patients' autopsies. The patients appeared in a coma and with convulsions after consuming an excessive amount of inexpensive red wine [4-6].

MBD is a rare degeneration of the CC that is caused by demyelination, necrosis, and occasionally hemorrhages, while the etiology is unknown [5]. MBD is primarily diagnosed in patients who drink alcohol chronically; however, it has also been seen in people who are malnourished or with frequent vomiting. Very rarely etiology of MBD has been attributed to toxins like organophosphorus poisoning [7]. Thiamine, vitamin B complex, and folate deficiencies are the nutritional deficiencies that link individuals with alcoholism and those diagnosed with malnutrition. Not every patient benefited, nevertheless, following supplementation [5].

Acute, subacute, or chronic neurological symptoms and signs can characterize the varied clinical presentation of MBD [5]. The abrupt onset of altered consciousness, convulsions, coma, and rapid progression to death characterize the acute presentation. The subacute appearance is typified by altered gait, mental disorientation, memory impairment, and behavioral abnormalities. The chronic form, which presents as gradual dementia with subtle changes over the years, is the least prevalent [1,2,5]. Brain MRI is the gold standard for diagnosing MBD [5]. CT scans are not sensitive enough to detect lesions at an early stage. Symmetric lesions in the CC are the typical MRI results, although lesions can also be seen in the brain, white matter, internal capsules, and middle cerebellar peduncles [8,9].

The whole appearance of our case took on significance since young pregnant females with MBD have not been recorded. This case report is significant for the management of patients in the antenatal period since hyperemesis gravidarum is a prevalent condition in pregnant women and may be a contributing factor to MBD.

It is important to take other disorders linked to alcohol into account when making a differential diagnosis for MBD. These include Wernicke's encephalopathy, multiple sclerosis, epilepsy, and neoplastic diseases. In contrast to MBD, which has a long recovery, Wernicke's encephalopathy typically presents with ataxia, visual dysfunction, such as nystagmus and ophthalmoparesis, disorientation, and therapy with thiamine results in quicker recovery.

Although a specific course of therapy is unknown, early detection and management with thiamine, folic acid, and B complex vitamins can hasten and improve healing. The goal of corticosteroid administration is to stabilize the blood-brain barrier and lessen inflammation [5]. MBD has a high fatality rate, and the acute variety frequently ends in death very fast. Severe neurological deficits are left behind in patients who survive [5]. Partial or full recovery can occasionally occur in certain patients with lesser signs [3]. A better prognosis is shown in patients who receive prompt treatment, have confined lesions on MRI, and have an early diagnosis [10]. The prognosis for MBD with alcoholic etiology is worse, with infective complications being the primary cause of mortality [10].

This case highlights the importance of suspecting rare acute demyelinating syndrome in a nutritionally deprived, non-alcoholic state which can be completely reversed with timely diagnosis and treatment with high-dose thiamine.

## Conclusions

MBD is a rare neurological condition that is typically linked to high death rates and excessive, prolonged alcohol usage. The diagnosis depends on the MRI's detection of necrosis and demyelination in regions with CC degeneration. The presentation of MBD in ANC patients presenting with chronic malnutrition and vomiting opens the door for clinicians to think in a vast array of directions. Although a particular therapy is not yet available, nutritional supplementation-especially with B complex vitamins-seems to support clinical improvement. Early diagnosis offers the best possibility of improving clinical outcomes, thus clinical suspicion is essential.

## References

[1] Tuntiyatorn L, Laothamatas J (2008). Acute Marchiafava-Bignami disease with callosal, cortical, and white matter involvement. Emerg Radiol.

[2] Ruiz-Martínez J, Martínez Pérez-Balsa A, Ruibal M, Urtasun M, Villanua J, Martí Massó JF (1999). Marchiafava-Bignami disease with widespread extracallosal lesions and favourable course. Neuroradiology.

[3] Carrilho PE, Santos MB, Piasecki L, Jorge AC (2013). Marchiafava-Bignami disease: a rare entity with a poor outcome. Rev Bras Ter Intensiva.

[4] Singh S, Wagh V (2022). Marchiafava Bignami disease: a rare neurological complication of long-term alcohol abuse. Cureus.

[5] Leong AS (1979). Marchiafava-Bignami disease in a non-alcoholic Indian male. Pathology.

[6] Kabra R, Patel M, Bhansali PJ, Kumar S, Acharya S (2022). Intermediate syndrome and Marchiafava-Bignami syndrome: double trouble in weaning off. Cureus.

[7] Tung CS, Wu SL, Tsou JC, Hsu SP, Kuo HC, Tsui HW (2010). Marchiafava-Bignami disease with widespread lesions and complete recovery. AJNR Am J Neuroradiol.

[8] Kohler CG, Ances BM, Coleman AR, Ragland JD, Lazarev M, Gur RC (2000). Marchiafava-Bignami disease: literature review and case report. Neuropsychiatry Neuropsychol Behav Neurol.

[9] Dong X, Bai C, Nao J (2018). Clinical and radiological features of Marchiafava-Bignami disease. Medicine (Baltimore).

[10] Hillbom M, Saloheimo P, Fujioka S, Wszolek ZK, Juvela S, Leone MA (2014). Diagnosis and management of Marchiafava-Bignami disease: a review of CT/MRI confirmed cases. J Neurol Neurosurg Psychiatry.

